# Application of Artificial Intelligence in Inborn Errors of Immunity Identification and Management: Past, Present, and Future—A Systematic Review

**DOI:** 10.3390/jcm14175958

**Published:** 2025-08-23

**Authors:** Ivan Taietti, Martina Votto, Marta Colaneri, Matteo Passerini, Jessica Leoni, Gian Luigi Marseglia, Amelia Licari, Riccardo Castagnoli

**Affiliations:** 1 Pediatric Unit, Department of Clinical, Surgical, Diagnostic, and Pediatric Sciences, University of Pavia, 27100 Pavia, Italy; ivan.taietti@gmail.com (I.T.); martinavotto@gmail.com (M.V.); gianluigi.marseglia@smatteo.pv.it (G.L.M.); amelia.licari@unipv.it (A.L.); 2 Pediatric Clinic, Fondazione IRCCS Policlinico San Matteo, 27100 Pavia, Italy; 3 Department of Infectious Diseases, ASST Fatebenefratelli Sacco University Hospital, 20157 Milan, Italy; marta.colaneri@gmail.com (M.C.); passerini.matteo@asst-fbf-sacco.it (M.P.); 4 Department of Pathophysiology and Transplantation, University of Milan, 20122 Milan, Italy; 5 Department of Electronics and Information of Politecnico di Milano, 20133 Milan, Italy; jessica.leoni@polimi.it

**Keywords:** inborn errors of immunity (IEI), primary immunodeficiencies (PIDs), artificial intelligence (AI), machine learning (ML), genetics, early diagnosis

## Abstract

**Background**: Inborn errors of immunity (IEI) are mainly genetically driven disorders that affect immune function and present with highly heterogeneous clinical manifestations, ranging from severe combined immunodeficiency (SCID) to adult-onset immune dysregulatory diseases. This clinical heterogeneity, coupled with limited awareness and the absence of a universal diagnostic test, makes early and accurate diagnosis challenging. Although genetic testing methods such as whole-exome and genome sequencing have improved detection, they are often expensive, complex, and require functional validation. Recently, artificial intelligence (AI) tools have emerged as promising for enhancing diagnostic accuracy and clinical decision-making for IEI. **Methods**: We conducted a systematic review of four major databases (PubMed, Scopus, Web of Science, and Embase) to identify peer-reviewed English-published studies focusing on the application of AI techniques in the diagnosis and treatment of IEI across pediatric and adult populations. Twenty-three retrospective/prospective studies and clinical trials were included. **Results**: AI methodologies demonstrated high diagnostic accuracy, improved detection of pathogenic mutations, and enhanced prediction of clinical outcomes. AI tools effectively integrated and analyzed electronic health records (EHRs), clinical, immunological, and genetic data, thereby accelerating the diagnostic process and supporting personalized treatment strategies. **Conclusions**: AI technologies show significant promise in the early detection and management of IEI by reducing diagnostic delays and healthcare costs. While offering substantial benefits, limitations such as data bias and methodological inconsistencies among studies must be addressed to ensure broader clinical applicability.

## 1. Introduction

Inborn errors of immunity (IEIs), previously known as primary immunodeficiencies (PIDs), are predominantly monogenic disorders that alter various components of the immune system, leading to altered immune functions and potentially life-threatening conditions [1]. IEIs are highly heterogeneous conditions characterized by a diverse combination of clinical manifestations, including severe and/or recurrent infections/or immune dysregulation, autoimmunity, hyperinflammation, lymphoproliferation, malignancies, and severe atopy. IEIs range from severe forms manifesting early in life, such as severe combined immunodeficiency (SCID), to milder (e.g., IgA deficiency) or more insidious forms that become evident later with prominent immune dysregulation or through susceptibility to specific pathogens (e.g., Mendelian susceptibility to mycobacterial disease, genetic susceptibility to herpes simplex virus 1 encephalitis) [2,3,4,5,6,7,8]. Moreover, several IEIs may also occur with a delayed onset and appear in adulthood, further complicating the diagnostic process [9].

Advances in understanding the molecular, cellular, immunological, and genetic mechanisms of IEI have improved patient outcomes, but timely and accurate diagnosis remains a significant challenge. Currently, no single assay can identify all types of IEI, which complicates screening efforts and underestimates the prevalence [10]. Even in countries with neonatal screening and IEI management programs, 70–90% of IEI cases remain undiagnosed, leading to adverse outcomes, including increased mortality [11,12].

IEIs pose a significant challenge to healthcare systems worldwide, requiring novel approaches for early diagnosis and effective management. Given a suspected clinical and immunological phenotype, next-generation sequencing (NGS), which includes whole-exome sequencing (WES) and whole-genome sequencing (WGS), represents the most potent confirmation tool, revealing molecular defects and gene-disease associations while providing insights into the disease mechanisms [13]. Despite the fact that achieving a molecular diagnosis offers a comprehensive understanding of IEI and allows for personalized surveillance strategies and interventions, NGS-associated costs are significant, and questions remain regarding the optimal timing and diagnostic yield. A key challenge in genetic testing is distinguishing pathogenic from non-pathogenic variants. WES and WGS often detect numerous rare variants, many of which are of unknown significance [14]. Although the onset of IEI is generally expected during childhood, the lack of IEI awareness among physicians and extensive screening programs may compromise the early diagnosis [15,16].

In this context, emerging computer technologies, particularly artificial intelligence (AI), may aid clinicians in identifying IEI and other rare diseases [17,18]. AI can uncover complex patterns and relationships within clinical, immunological, and microbiological data, using genetic data as a label to train the model and evaluate its performance in predicting IEI. The promising outcomes of these techniques are already evident in Clinical Immunology, where computational algorithms have proved to be practical tools for diagnosing and tracking patients with IEI [19,20]. Machine learning (ML) is a data-driven analytic approach that identifies a model by analyzing underlying patterns in data [21,22]. Specifically, ML algorithms integrate complex data sources, providing valuable support to physicians in the decision-making process. Thus, integrating AI in clinical-decision support systems (CDSSs) would lead to tools that allow for advanced genomic analysis in a comprehensive way, considering clinical, immunological, and microbiological data, to detect at-risk patients, plan personalized strategies, identify patterns in disease to facilitate new genetic discoveries, and reduce diagnostic time to recognize IEI [23,24,25,26,27]. These innovative approaches might serve as essential tools for healthcare professionals, enabling early detection and personalized management strategies for patients at risk of having IEI. Providing evidence-based recommendations will empower clinicians to make informed decisions, ultimately improving patient outcomes. In addition, if adequately designed, AI-based tools might deepen the understanding of IEI pathogenesis, identify novel genetic variants, and contribute to the development of targeted therapies, thereby improving the life expectancy of IEI patients. Moreover, AI technology might reduce the healthcare costs associated with WES/WGS analysis and interpretation. The growing availability of genetic data opens new avenues for precision diagnostics. AI may also play a critical role in interpreting this complex information and supporting early, data-driven decision-making.

This review examines the current AI-based approaches in IEI patient care, assessing their ability to facilitate IEI identification. We evaluate the potential for a cost-effective strategy that integrates clinical, immunological, and microbiological data while reserving genomic analyses for cases where they are most beneficial. Overall, our review outlines IEI pathology, current diagnostic approaches, and the expected advancements in early detection, targeted intervention, and improved quality of life for patients through AI applications.

## 2. Materials and Methods

The protocol of this review was registered (ID registration CRD42024547341) and published with the International Prospective Register of Systematic Reviews (PROSPERO, https://www.crd.york.ac.uk/prospero/display_record.php?ID=CRD42024547341, accessed on 16 May 2024) before the study started.

### 2.1. Inclusion Criteria and Search Strategy

An extensive search strategy was designed to retrieve all articles published up to 16 May 2024 (Table 1). An electronic search was conducted in four databases (PubMed, Scopus, Web of Science, and Embase) in accordance with the Preferred Reporting Items for Systematic Reviews and Meta-analyses (PRISMA) reporting guidelines [28] (Table S1).

We used the following Medical Subject Heading (MeSH) terms: (“Primary immunodef*” OR “Inborn error of immunity”) AND (“artificial intelligence” OR “machine learning”). All studies meeting the following criteria were included: (i) original research articles (retrospective or prospective studies, clinical trials) published in English in peer-reviewed journals; (ii) participants were children and adult patients with a diagnosis of IEI according to Human IEI International Union of Immunological Societies (IUIS) Expert Committee Classification [1]; (iii) application of AI or ML models to improve diagnosis and knowledge of IEI molecular signature and pathogenesis.

### 2.2. Data Extraction

Two investigators (I.T. and M.V.) independently scrutinized the eligibility of the identified titles and abstracts based on the elements of the “application of any type of AI tools in IEI’s field” question. The same investigators independently assessed full texts of records deemed eligible for inclusion. A third author (R.C.) resolved disagreements between the first two authors to reach a consensus. The Rayyan^R^ software (https://rayyan.ai/cite?_ga=2.168307361.1602442765.1755690718-1866348700.1755690718, accessed on 17 August 2025) was used to screen articles. Two independent reviewers (I.T. and M.V.) extracted data from each eligible study using a standardized Excel spreadsheet and then proceeded to cross-check the results. Disagreements between reviewers regarding extracted data were resolved through discussion and consensus of a third reviewer (R.C.). The following information was extracted: first author name, date of publication, type of study (retrospective or prospective study, clinical trials), number of patients, IEI type, age (children, adults), AI models, measures of effect (area under the curve, sensitivity, specificity, and accuracy), and outcome. Detailed information on the included studies is provided in a summary table (Table 2).

Risk of bias assessment. The risk of bias and applicability of eligible studies were assessed using the Prediction Model Study Risk of Bias Assessment Tool (PROBAST) [29,30]. The risk of bias and applicability are classified as low, unclear, or high. The evaluation tool contains 20 signaling questions from four domains: participants, predictors, outcomes, and analyses. Two investigators (I.T. and M.V.) independently assigned an overall risk of bias to each eligible study, and if they disagreed, a third reviewer (R.C.) was consulted.

**Table 2:** Summary of the included studies.

Author, Year	Number of Patients	AI Type	AUC	Sensitivity	Specificity	Accuracy	Error	IEI	Outcome
Keerthikumar et al., 2009 [31]	Training dataset: - Positive dataset (all the known PID genes): 148 genes. - Negative dataset (genes with no immune/hematopoietic system abnormalities): 3162 genes.	SVM learning	/	0.85	0.98	/	Training data set: - LOO error: 8–15%	Myd88 deficiency PRKCD deficiency *G6PC3* mutations ITK deficiency *CORO1A* mutations	Predict candidate PID genes
	Validation/Test dataset: 36,677 genes.						/		
Woellner et al., 2010 [32]	Training dataset: 100	SVM learning	/	Training dataset: 0.88	Training dataset: 0.81		Training dataset: - LOO error: 15%	*STAT3* AD HIES	Clinical assessment of HIES prior to a confirmation of the suspected diagnosis by laboratory and molecular analysis (i.e., the presence of a *STAT3* mutation).
	Validation/Test dataset: 50			Validation/Test dataset: 0.94	Validation/Test dataset: 0.71		Validation/Test dataset: - LOO error: 14%		
Orange et al., 2011 [33]	Training data set: - CVID patients: 179 - healthy controls: 1197	SVM learning	/	/	/	/	*/*	CVID	Link SNP with likelihood for CVID from GWAS for an earlier diagnosis.
	Validation/Test dataset: - CVID patients: 109 - Healthy controls: 1114		/	/	/	Validation/Test dataset: 0.99	/		
Engelhardt et al., 2015 [34]	Validation/Test dataset: 82	SVM learning	/	0.91	0.88	/	11.1%	*DOCK8* AR HIES	Compare clinical data from patients with DOCK8 deficiency with AR-HIES patients without a *DOCK8* mutation and patients with *STAT3* mutations.
Mücke et al., 2017 [35]	Training set: 99	SVM learning, RF, LR, BN, linear discriminant analysis and nearest neighbor classifiers	/	Training dataset: 0.98	/	/	/	CVID Ataxia teleangiectasia C2 deficiency Cernunnos CID with different cytopenia Nijmegen breakage syndrome X-linked CGD Undefined severe immunodeficiency	To support physicians to consider a PID with a questionnaire based on patient’s experience.
	Validation/Test set: 27			Validation dataset: 0.90	/	/	/		
Emmaneel et al., 2019 [36]	Validation/Test set: 179	Machine learning (SVM and RF)	/	/	/	0.93	/	CVID	To assist the diagnosis of CVID patients and differentiate them from other PADs.
Berbers et al., 2021 [37]	Training set: 45	Machine learning (RF, Enet, XGboost)	Training set: - RF: 0.71 - Enet: 0.72 - XGboost: 0.70	Training set: - RF: 0.70 - Enet: 0.74 - XGboost: 0.78	Training set: - RF: 0.71 - Enet: 0.71 - XGboost: 0.67	/	/	CVID	To assess the potential of serum proteomics in the stratification of patients with immune dysregulation and to identify cytokine and chemokine signaling pathways underlying immune dysregulation in CVID.
	Validation/Test set: 74		Testing cohort: - Enet: 0.73	Testing cohort: - Enet: 0.83	Testing cohort: - Enet: 0.75		/		
Rider et al., 2021 [23]	Training set: 100	BN probabilistic model (“PI prob”)	Validation/Test set: 0.95	Validation/Test set: 0.87	Validation/Test set: 0.91	Validation/Test set: 0.89	/	CID PADs Immune disregulatory disorder Phagocyte disorders Innate disorders AID Complement disorders	To predict risk of primary immune defects and guide clinical decision-making process.
	Validation/Test set: 75						/		
Diana et al., 2022 [38]	Training set: 32	BN	/	At 3 months: 0.41. At 6 months 0.74.	At 3 months: 0.88. At 6 months 0.90.	At 3 months: 0.79. At 6 months 0.87.	Relative standard error: 8.03–9.93	SCID	To explore a modelling approach for the prediction of the time course and extent of CD4^+^ T-cell immune reconstitution after SCID transplantation.
Abyazi et al., 2021 [39]	79 patients: - CVID patients: 26 subjects with complicated CVID and 31 subjects with uncomplicated CVID; - Control patients: 12 age and sex-matched HCs and 10 subjects with other forms of PAD	MFA	/	/	/	/	/	CVID	Identify, correlate, and determine the significance of immunologic features of CVID with non-infectious complications.
Fang et al., 2022 [14]	Training dataset: - IEI SNVs: 4865 - non-IEI SNVs: 4237	ML (Conditional Inference Forest model)	Training set: - Non-gene-specific: 0.89 - Gene-specific: 0.91	/	/	/	/	Immune dysregulation Syndromic CID PAD Complement defects CID Phagocytic defects AID	Predict the pathogenicity of SNVs for IEI, using a gene-specific approach through the functional validation of SNVs in IEI genes with improved accuracy.
	Validation/Test dataset: 1318 patients		Validation set: 0.86				/		
Takao et al., 2022 [40]	Training set: 102 patients	LR and ML (RF, XGboost and CART)	Training set: /	Training set: /	Training set: /	Training set: /	/	SIgAD Isolated primary neutropenia THI, History of severe infections with lymphopenia awaiting lymphocyte subsets counting Glycogen storage diseases with associated neutropenia CVID CGD Cyclic neutropenia DiGeorge Syndrome WAS	Measure the individual chance of a confirmed diagnosis of IEI in children at risk, according to previous evaluation by general pediatrician/ Clinician.
	Validation/Test set: 26 patients		Validation/Test set: - RF:0.94 - XGboost:0.85 - CART: 0.87 - LR: 0.80	Validation/Test set: - RF: 0.95 - XGboost: 0.86 - CART: 0.82 - LR: 0.77	Validation/Test set: - RF: 0.63 - XGboost: 0.56 - CART: 0.69 - LR: 0.63	Validation/Test set - RF: 0.93 - XGboost: 0.84 - CART: 0.85 - LR: 0.79	/		
Mayampurath et al., 2022 [41]	Training set: - Case set (IEI): 247; - Control set (asthma): 6175)	LR, EN, RF	- LR: 0.70 - EN: 0.70 - RF: 0.62	- LR: 0.79/72 - EN: / - RF: /	- LR: 0.58/0.62 - EN: / - RF: /	/	/	DiGeorge syndrome CVID CID SCID WAS Congenital hypogammaglobulinemia Evans Syndrome ALPS	To develop a predictive algorithm for early diagnosis of IEI using EHR data and ML.
Rider et al., 2022 [27]	Training set: 428 patients	ML (SVM, LR, DNN)	/	/	/	/	/	IEI with other major disease PAD Complement disorders and other IEI CVID Disorders of neutrophils CID	Population-wide detection of infection susceptibility and risk of IEI.
	Validation/Test set: 184		Validation/Test set: - SVM: 0.52 - LR: 0.97 - DNN 1 × 128: 0.98 DNN 2 × 128: 0.99 - DNN 1 × 64: 0.95	/	/	Validation/Test set: - SVM: 0.87 - LR: 0.98–0.99 - DNN 1 × 128: 0.99 DNN 2 × 128: 0.99 - DNN 1 × 64: 0.96	/		
Ding et al., 2023 [42]	Training set: 129 (no-PID:PID = 75:54)	LR	Training set: 0.84	Training set: 0.78	Training set: 0.78	Training set: 0.72	Training set: 4%	/	To use the analysis of radiomics based on un-enhanced CT for identifying immunodeficiency status in children with PTB.
	Validation/Test set: 44 (no-PID:PID = 26:18)		Validation set: 0.85	Validation/Test set: 0.83	Validation/Test set: 0.72	Validation/Test set: 0.68	Validation/Test set: 7.4–14%		
Wang et al., 2023 [43]	Training set: 152 patients	Multivariate LR, Lasso, RF, XGboost	Training set: 0.83	Training set: 0.77	Training set: 0.77	/	/	SCID VEO-IBD CGD WAS LAD HIGM syndrome	Identify pre-transplantation risk factors associated with early mortality and build predictive models through ML algorithms in pediatric IEI patients who underwent UCBT.
	Validation/Test set: 78 patients		Validation set: 0.74	Validation set: 0.54	Validation set: 0.82	/	/		
Méndez Barrera et al., 2023 [18]	Training set: 1677	XGBoost	Training set: - CVID: 0.80	/	/	Training set: - CVID: 0.80	/	CVID DGS Congenital agammaglobulinemia Not otherwise classified PID PAD CGD Complement deficiency HIGM syndrome LAD Ectodermal dysplasia with immune deficiency SCID WAS	Predict IEI
	Validation/Test set: 719		Validation set: - CVID: 0.75 - DGS: 0.87 - Congenital agammaglobulinemia: 0.76 - Not otherwise classified PID: 0.74 - PAD: 0.74 - CGD: 0.83 - Complement deficiency: 0.46 - HIGM syndrome: 0.65 - LAD: 0.66 - Ectodermal dysplasia with immune deficiency: 0.62 - SCID: 0.73 - WAS: 0.74	/	/	Validation set: - CVID: 0.76 - DGS: 0.90 - Congenital agammaglobulinemia: 0.89 - Not otherwise classified PID: 0.88 - PAD: 0.84 - CGD: 0.92 - Complement deficiency: 0.92 - HIGM syndrome: 0.93 - LAD: 0.99 - Ectodermal dysplasia with immune deficiency: 0.97 - SCID: 0.85 - WAS: 0.76	/		
Lu et al., 2024 [17]	- Training set: 60 - Validation set: 133 - Test set: 186	AI-assisted workflow (DeepFlow^TM^)	/	/	/	/	/	Not specified	To diagnose immunological disorders in a clinical setting through AI-assisted flow cytometry providing a transformative approach within a concise timeframe.
Roberts et al., 2024 [44]	Training set: 5.901	Linear SVM	0.95	/	/	/	/	Hypogammaglobulinemia DGS SIgAD CID	To develop an NLP system capable of early IEI patient recognition using real-world clinical notes before their receipt of an IEI diagnosis via clinical practice.
Rider et al., 2024 [45]	Validation/Test set: - IEI cohort: 41,080 - Control cohort: 250,262	SPIRIT analyzer (rule-based AI tool)	/	/	/	/	/	CID CID with syndromic features PAD Disorders of immune dysregulation Phagocyte defects Innate immune disorders AID Complement disorders	Quantify the value of having >2 WS for the diagnosis of IEI using a highly representative real-world US cohort.
Johnson et al., 2024 [46]	- Training set: 197 (CVID) vs. 1106 (control) - Validation/Test set: 100 (CVID) vs. 100 (control)	PheNet (marginal LR)	- Training set: AUC-ROC: 0.95 AUC-PR: 0.96	/	/	/	/	CVID	To recognize clinical features of patients with CVID from their EHR data identifying undiagnosed patients.
Sobrino et al., 2024 [47]	5 patients	Elastic-net LR	0.99	/	/	0.97	/	XL-CGD	To identify predictive markers of engraftment failure in patients with XL-CGD who underwent GT.
Pello et al., 2024 [48]	Validation/Test dataset 1: 32 Validation/Test dataset 2 (naïve T-cell available): 24 [80% of samples for model training and 20% of samples for model validation]	RF	/	/	/	Validation/Test dataset 1 (training set): - Respiratory infections: 0.93 - Severe infections: 0.93 Validation/Test dataset 2 (training set): - Respiratory infections: 1 - Severe infections: 1	/	Non–severe CID CHH	To correlate early childhood clinical and laboratory features with clinical outcomes on long-term follow-up of CHH patients.

AD, autosomal dominant. AI, artificial intelligence. AIDs, autoinflammatory diseases. ALPS, autoimmune lymphoproliferative syndrome. AR, autosomal recessive. AUC, area under the curve. BN, Bayesian network. BP, branchpoint. CART, classification and regression tree. CHH, cartilage–hair hypoplasia. EN, elastic nets. CGD, chronic granulomatous disease. CID, combined immunodeficiency. CT, computed tomography. CVID, common variable immune deficiency. DGS, DiGeorge syndrome. DNN, dense neural network. DOCK8, dedicator of cytokinesis 8. EHR, electronic health records. Enet, elastic net regression. GT, gene therapy. GWAS, genome-wide association. HIGM, hyper-IgM. HIES, hyper-IgE syndrome. HSCT, hematopoietic stem cell transplantation. ID, immune deficiency. HPO, human phenotype ontology. IEI, inborn errors of immunity. LAD, leukocyte adhesion deficiency. LOO error: leave-one-out error. LR, logistic regression. MFA, multiple factor analysis. ML, machine learning. NLP, natural language processing. NOS, not otherwise specified. PADs, predominantly antibody deficiencies. PID, primary immune deficiency. RF, random forests. SCID, severe combined immunodeficiency. SIGSD, IgG subclass deficiency. SIgAD, selective IgA deficiency. SIgMD, selective IgM deficiency SNP, single-nucleotide polymorphism. SNV, single-nucleotide variant. SPIRIT, software for primary immunodeficiency recognition, intervention, and tracking. STAT3, signal transducer and activator of transcription 3. SVM, support vector machine. THI, transient hypogammaglobulinemia of infancy. UCBT, umbilical cord blood transplantation. US, United States. VEO-IBD, very early onset inflammatory bowel disease. WAS, Wiskott–Aldrich syndrome. WS, warning signs. XGboost, extreme gradient boosting machine model. XL, X-linked.

## 3. Results

**Figure 1:**
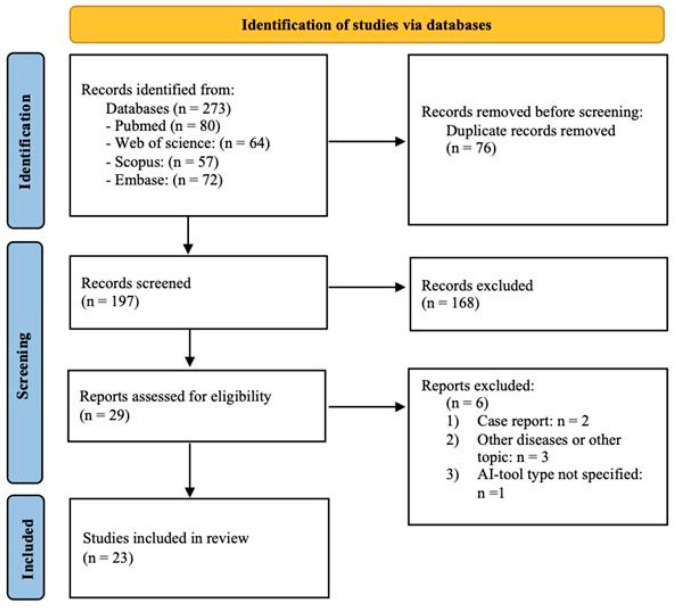
Study flowchart from identification to inclusion of final articles.

We found a total of two hundred seventy-three (273) articles. After seventy-six (76) duplicates were removed and one hundred ninety-seven (197) articles were screened, one hundred fifty-two (152) were selected based on their titles and abstracts. A total of twenty-nine (29) studies were assessed for eligibility (Figure 1), and twenty-three (23) articles met the inclusion criteria and were included in the final review (Table 2).

### 3.1. AI and IEI Genetics

Keerthikumar et al. developed a support vector machine (SVM) learning algorithm to predict candidate IEI genes [31]. The model was trained on 69 features across two datasets: positive (known IEI genes, totaling 148) and negative (genes with no immune or hematopoietic impact, totaling 3162). It achieved a sensitivity of 0.85 and a specificity of 0.98, identifying 1442 potential PID genes. It accurately predicted genes confirmed as IEI-associated but not initially included in the training group [myeloid differentiation factor-88 (MYD88), the catalytic subunit of DNA dependent serine/threonine protein kinase (PRKDC), glucose-6-phosphatase, catalytic subunit 3 (G6PC3), IL2-inducible T-cell kinase (ITK), coronin, actin binding protein 1A (CORO1A), and Interleukin 1 receptor antagonist (IL1RN)].

Orange et al. trained and validated an SVM to predict common variable immune-deficiency (CVID) phenotypes from single-nucleotide polymorphisms (SNPs) and copy number variations (CNVs) [33]. The model, trained on 1000 SNPs, distinguished CVID cases from controls with 98.7% accuracy and displayed predictive values of 1.0 and 0.957, supporting the genetic heterogeneity of CVID and aiding in diagnosis.

Fang et al. developed a variant impact predictor for PID (VIPPID), a disease-specific tool that predicts the pathogenicity of SNVs for IEI [14]. This tool uses a gene-specific approach, training sub-models for each common IEI gene. VIPPID can predict the functional consequences of SNVs in IEI genes with high accuracy (training set: non-gene-specific, 0.89; gene-specific, 0.91; validation set, 0.86), distinguishing PID-related mutations from non-disease-causing mutations.

### 3.2. AI in the Real-World Clinic of Patients with IEI

Woellner et al. applied SVM to classify suspected hyper-IgE syndrome (HIES) cases, examine genotype-phenotype correlation, and analyze signal transducer and activator of transcription 3 (*STAT3*) sequencing [32]. They achieved 87.5% sensitivity and 80.6% specificity in the training set for using the clinical and biochemical features to predict the genotype of the *STAT3* mutation. These features include a high level of serum IgE (>1000 IU/mL), recurrent pneumonia, newborn rash, pathologic bone fractures, facial characteristics of Job’s syndrome, palate structure (i.e., high palate), lack of T-helper (Th) 17 cells, and positive family history. This novel approach helped to refine diagnostic guidelines for HIES.

Engelhardt et al. used an SVM to distinguish patients with dedicator of cytokinesis 8 (DOCK8) deficiency from those with STAT3 deficiency or other immune disorders [34]. They reduced the National Institute of Health (NIH)’s 20 clinical features to five key indicators, including (i) lung abnormalities, (ii) eosinophilia, (iii) upper respiratory infections, (iv) retained primary teeth, and (v) fractures with minimal trauma, achieving a sensitivity of 91.4% and specificity of 87.5% [with eosinophilia and upper respiratory infections (sinusitis and otitis) more prevalent in DOCK8 deficient patients, while the others more prevalent in STAT3 deficiency].

Pre-diagnostic experiences of IEI patients could help reach an earlier diagnosis. Therefore, Mücke et al. developed a 36-item questionnaire using combined AI tools [35]. During the first step of the analysis, the diagnostic accuracy was challenged after 99 individuals answered two sets of questionnaires (64 children with IEI and 35 healthy controls), with an overall sensitivity of 98%. During the second step, the system was tasked with distinguishing between 127 questionnaires from children with IEI and a mix of randomly selected healthy children and those suffering from various illnesses (e.g., severe bronchitis, brain tumor, cystic fibrosis, and ulcerative colitis). This step achieved an overall sensitivity of 90%.

Rider et al. built a predictive model based on history, physical examination, laboratory data, and general and highly informative infections or conditions [23]. The BN model provided a real-time risk assessment of PID and facilitated prescriptive analytics to determine the most appropriate diagnostic work-up. The model enabled accurate and objective decision-making about risk, guiding the user toward the proper diagnostic evaluation for patients with recurrent infections. This resulted in improved diagnostic rates for IEI and a shorter time to diagnosis, with higher performance compared to other ML models.

Diana et al. investigated a BN modeling approach derived from a mixed-effects framework and early post-hematopoietic stem cell transplantation (HSCT) data to predict the time course and the extent of CD4^+^ T-cell immune reconstitution in SCID patients [38]. Genetic diagnosis and conditioning regimen (CR) were found to be the two pre-transplant variables significantly associated with the time course of T-cell reconstitution. Patients with RAG1 or ARTEMIS deficiency, as well as those with other diagnoses, exhibited a 36.8% decrease in their thymic output function compared to those with an IL2RG/JAK3 defect. Moreover, CR significantly decreased the cell loss rate function. The dose of anti-thymocyte globulin (ATG), immediate pre-HSCT morbidities, or the stem cell graft’s composition did not substantially influence T cell reconstitution.

Takao et al. developed and compared logistic regression (LR) and ML models to predict IEI risk, thus helping general pediatricians and clinicians in screening IEI [40]. This approach reduced the time between diagnosis and treatment, complications, and overall care costs. They demonstrated that variables not included in the Jeffrey Modell Foundation 10 Warning Signs for Immunodeficiency (JMF10WS), such as IgA, IgM, and IgG levels, lymphopenia, body weight, and age, were associated with a greater likelihood of having IEI.

Patients typically undergo various treatment approaches before receiving a definitive diagnosis of IEI. This information can be used to recognize patients early, thus reducing diagnostic delay and ensuring timely treatment. Mayampurath et al. trained and validated an AI model using electronic health records (EHRs) data [i.e., (i) comorbidities, (ii) laboratory tests, (iii) radiological orders, and (iv) medications] from a patient’s historical interaction with a hospital for early diagnosis of patients with IEI, demonstrating the superiority of the LR-based tool upon RF and (Enet)-based ones [41].

Rider et al. built and validated a generalizable analytical pipeline for population-wide detection of infection susceptibility and risk of IEI, trained, and compared different ML approaches [LR, RF, dense neural network (DNN), and SVM] [27]. They started with categorizing through the software for primary immunodeficiency recognition, intervention, and tracking (SPIRIT) analyzer. Patients initially classified as high risk by SPIRIT showed a new diagnosis rate of 9%, compared to only 0.2–1.5% in other risk categories.

Wang et al. developed and validated an ML algorithm to predict early (first 50 days) all-cause mortality after umbilical cord blood transplantation (UCBT) in patients with different IEIs [SCID, very early onset inflammatory bowel diseases (VEO-IBD), chronic granulomatous disease (CGD), Wiskott–Aldrich syndrome (WAS), leukocyte adhesion deficiency (LAD), hyper-IgM (HIGM) syndrome] based on pre-transplant risk factors [43]. Pretransplant albumin, absolute count of CD4^+^ cells, elevated C-reactive protein, and history of sepsis predict early mortality with high performance in both training and validation sets (training set: AUC 0.83, sensitivity 0.77, and specificity 0.77; validation set: AUC 0.74, sensitivity 0.54, and specificity 0.82).

Méndez Barrera et al. realized an extreme gradient boosting ML model (XGBoost)-based algorithm based on clinical and laboratory features to predict any out of 12 IEI diagnoses [(i) CVID, (ii) DiGeorge syndrome (DGS), (iii) congenital agammaglobulinemia, (iv) not otherwise classified PID, (v) predominantly antibodies deficiencies (PAD), (vi) CGD, (vii) complement deficiency, (viii) HIGM syndrome, (ix) LAD, (x) ectodermal dysplasia with immune deficiency, (xi) SCID, and (xi) WAS], using a dataset of over 2300 patients from the United State Immunodeficiency NETwork (USIDNET) registry. The model showed a good performance [the area under the curve (AUC) was 0.70–0.80, and accuracy 0.75–0.90] and precision in recognizing WAS, DGS, and CVID [18].

Natural language processing (NLP) is a computer science branch that enables computers to interpret unstructured, free-text documents. Roberts et al. built and trained a natural NLP SVM-based algorithm with temporal fidelity that permits disease recognition up to 36 months before clinical IEI diagnosis with an area under the precision–recall curve > 0.95 [44].

Riders et al. applied a SPIRIT analyzer to screen individuals at risk for IEI and facilitate early diagnosis for a large cohort of US patients from the EHR [45]. They demonstrated that the presence of WES and associated clinical diagnoses can facilitate the identification of patients with IEI from EHR data.

Johnson et al. showed that ML algorithms (i.e., PheNet based on marginal LR) can learn from the EHR to expedite the diagnosis of patients with CVID and to identify phenotypic patterns of rare diseases [46]. The model utilized 44 phecodes (i.e., simplified categorization of diagnoses compared to the International Classification of Diseases (ICD) codes) and IgG levels to generate a risk score for each patient. This approach enabled the identification of patients with CVID 1 to 4 years before diagnosis.

Sobrino et al. applied Enet LR to five patients with CGD who underwent gene therapy [47]. They identified a set of 51 interferon (IFN) genes and transcription factors as predictive markers of the HSC engraftment failure. Therefore, relevant therapeutic consideration that targeted treatments to protect HSC, coupled with targeted gene expression screening, might improve clinical outcomes in CGD.

Pello et al. validated an RF-based tool to detect infections in children with non-severe cartilage-hair hypoplasia (CHH) combined immunodeficiency (CID) [48]. The choice of this model also enhances interpretability, as it enables the ranking of feature importance after training, providing valuable insights into the decision-making process. The results indicate that shorter birth length, naive T-cell count, and function reduction are the most important features associated with the development of the disease, providing physicians with a deeper understanding of this condition.

### 3.3. AI and Immunological Investigation

Emmaneel et al. used an automated ML pipeline (comparing SVM-based and RF) that performs automated diagnosis based on flow cytometric immunophenotyping to expedite the diagnosis of CVID patients, distinguishing them from other PAD [36]. The computational time resulted in a considerable reduction compared to the manual method, demonstrating a significant advantage of using ML-derived models during the diagnostic process for patients. Among the evaluated approaches, flowSOM features proved to be faster and more accurate than models based on manually gated cell populations, with superiority of SVM over RF. The model provided a deeper insight and offered a more specific marker profile of immune cells, including the following:

- The percentages of switched memory B cell IgG^+^, CD38^+^, and CD21^+^ decreased, and the percentages of switched memory B cell CD21^+^, CD24^+^, and IgA^+^ increased.
- The increase in FoxP3 for a specific regulatory T cell population and CCR7 for another regulatory T cell population.
- The increase in the CD14 markers for the conventional dendritic cell population.
- The increase in CD8^+^ effector memory T cells and CD8^+^CD45RO-CD31^+^ T cells.
- The decrease in specific gamma delta T cells in the CVID patients.

Berbers et al. used ML models (RF, Enet, XGboost) to stratify CVID patients with immune dysregulation through serum proteomics and identify cytokine and chemokine signaling pathways underlying immune dysregulation in CVID [37]. Within a total of 180 serum inflammation and immune response-related proteins, mast cell immunoglobulin-like receptor 1 (MILR1), leukocyte immunoglobulin-like receptor subfamily B member 4 (LILRB4), IL-10, IL-12 receptor subunit beta 1 (IL-12RB1), tumor necrosis factor receptor superfamily member 9 (TNFRSF9) and CD83 (an immunoglobulin superfamily receptor expressed by mature antigen-presenting cells) were selected as the most important markers. The Enet model yielded the best combination of high performance (AUC 0.73, sensitivity 0.83, and specificity 0.75). IL-10, IL-12RB1, and CD83 were significantly upregulated in CVID-immune dysregulation (CVIDid) compared to CVID-infections only (CVIDio) in both the training and testing cohorts. These results provided a promising first step in the development of a screening tool for immune dysregulation in CVID. Furthermore, the immune dysregulation phenotype was associated with elevated levels of Th1- and Th17-related serum proteins, exhibiting a complex immune regulatory profile involving IL-10, LAG3, TNFRSF9, and CD83 signaling pathways.

Abyazi et al. applied multiple factor analysis (MFA) to the data of plasma cytokines and chemokines, total and LPS-specific antibody responses, and peripheral blood leukocyte immunophenotyping from 57 subjects with CVID [complicated (CVIDc) and uncomplicated] [39]. Different “traits” were identified, specifically: (i) elevated IL-6, IL-18, and IFNγ; (ii) increased soluble IL-18 receptor, colony-stimulating factor 1, lymphotoxin alpha, LIGHT, oncostatin M, TNF, and vascular endothelial growth factor A; (iii) elevation of IL-12p40, the common subunit of IL-12 and IL-23; (iv) elevations of 4-1BB, CD40, and T-cell chemoattractants; (v) increase in CD5, CD6, and FMS-like tyrosine kinase 3 ligand; (vi) increased plasma sCD14; (vii) reduced levels of IgA as well as LPS-specific IgA and IgM. This unsupervised ML algorithm validated the association of elevated plasma cytokines with more significant antibody defects and skewed T-cell immunophenotype. These results demonstrated that convergence of these traits could accurately differentiate subjects with CVID.

Lu et al. developed and validated an AI-assisted flow cytometry workflow utilizing the DeepFlowTM software (version 2.1.1) [17]. They introduce a simple, efficient, and automatic AI-assisted system that standardizes and achieves high-quality flow results, leading to accurate diagnoses and increased productivity. Thus, the system can potentially increase laboratory workflow efficiency and enhance data interpretation consistency.

### 3.4. AI and Radiology Are Unveiling the Potential Underlying IEI

Ding et al. demonstrated that radiomics combined with an LR model can identify the immunodeficiency status in children with pulmonary tuberculosis, aiding in the detection of PID [42].

### 3.5. Risk of Bias Assessment

**Figure 2:**
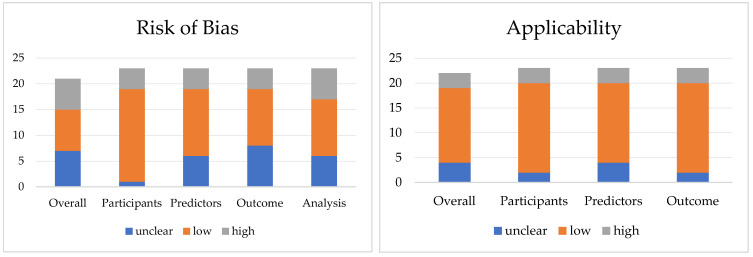
Risk of bias assessment according to PROBAST.

Using PROBAST, 8 (35%) and 15 (65%) studies showed a low risk of bias and applicability, respectively. Seven (30%) and six (26%) studies were classified as having unclear and high risk of bias, respectively, and four (17%) and three (13%) studies were classified as having unclear and high concern about applicability, respectively. Considering the single domains analyzed, for “participants,” most studies were classified as having a low risk. Concerns about applicability were classified as low for the “participants,” “outcome,” and “predictors” domains. However, the high risk of bays resulted from the “analysis” domain, while high concern about applicability arose from all three domains analyzed (Figure 2).

## 4. Discussion

The use of AI and ML in diagnosing and managing IEI represents a significant advance in clinical immunology. Given the high heterogeneity of IEI and their complex genetic underpinnings, traditional diagnostic processes are hindered by variability in clinical presentations, leading to diagnostic delays despite clinicians’ best efforts. IEIs are a highly heterogeneous group of rare and mainly monogenic diseases caused by increasingly identified genetic mutations. Several unmet diagnostic and therapeutic needs still exist, and genetic diagnosis is not always done [1,49,50]. No uniform clinical presentation exists, and diagnostic variability remains extensive. Despite the best efforts of clinical immunologists, significant delays in diagnosis persist [51].

Nowadays, AI and ML-based tools are applied in several clinical immunology fields, becoming integral for predicting, diagnosing, and managing IEI. The power of AI lies in its ability to use large datasets to discover patterns and extract statistical rules that enable it to make reliable predictions about new data belonging to the same distribution. Table S2 summarizes the main features of AI tools reported in the systematic review. These tools allow the early detection and accurate diagnosis of genetic mutations, clinical features, SNPs, and biomarkers with greater precision and speed than traditional methods. Early diagnosis is essential for patients and may also have a financial impact in terms of longer-term savings by reducing complications. Due to the flexibility and generality of these approaches, ML has diverse applications in IEI diagnosis, ranging from genetic prediction models to CDSS based on EHR data and automated flow cytometry analysis [14,27,41]. This result demonstrates that the ML filter enables more fine-grained risk assessments, allowing for the scaling of interventions and driving cost savings by preventing unnecessary evaluations and low-yield testing. Therefore, the application of AI in IEI supports the findings of a recent systematic review that evaluated the potential of AI in reducing healthcare-related costs, especially those associated with unnecessary diagnostic tests and inappropriate treatment [52].

SVM is a powerful ML technique widely used in computational biology [53]. Algorithms like SVM were used to predict potential IEI genes with high sensitivity and specificity by analyzing gene data [31]. SVM-based approaches have improved the early recognition of IEI, thanks to the combination of genetic mutations and clinical features [32], also enhancing the precision in the differential diagnosis of DOCK8 and STAT3 deficiencies [34]. Moreover, SVMs have been utilized to identify SNPs and CNVs, as well as to predict CVID phenotypes. This model can differentiate between the phenotypes of diseases and healthy controls with high accuracy, helping clinicians make faster and more accurate diagnoses in clinical practice, especially in cases with heterogeneous phenotypes, such as CVID [33]. Moreover, VIPPID represents another tool for predicting the pathogenicity of SNVs specific to IEI genes, thereby improving diagnostic precision [14].

To enhance the clinical care of CVID patients, there is a critical need to identify predictive biomarkers for immune dysregulation and those that can monitor therapeutic responses. Thus, a deeper understanding of the immune mechanisms driving dysregulation could reveal new therapeutic targets and improve the selection of innovative targeted immunotherapies. RF-based and MFA models identify biomarkers to distinguish between CVID phenotypes and immune dysregulation, thus improving patient stratification and treatment outcomes [37,39]. RF demonstrated its superiority among ML models trained and validated by Takao et al. for predicting IEI risk, thereby helping pediatricians/clinicians in patient screening [40]. Moreover, an RF-based tool eases the interpretability and explainability of the decision-making process. Accordingly, Pello et al. effectively identified key features associated with the development of infection in children with non-severe CHH CID. They ranked their significance in decision-making upon relative importance thanks to the combination of traditional statistical analyses and ML. This method lets them address the challenges of small sample sizes and variations in the availability and distribution of laboratory data [48].

NLP, ML, and computer-aided diagnostic algorithms can effectively analyze large datasets, including EHRs and data warehouses, for high-quality and precise care [54,55,56,57,58]. They also inform CDSS, facilitating the use of EHRs for direct patient care [59,60,61,62]. In several studies, ML models that integrate AI-based questionnaires and EHR were used to predict and preemptively diagnose IEI based on patient history and clinical data, thus significantly reducing diagnostic time and improving patient outcomes. AI-driven questionnaires demonstrate how simple tools can enhance diagnostic accuracy [35]. PheNet has shown high performance in recognizing CVID clinical features, thereby expediting diagnosis and the identification of phenotypic patterns, highlighting the importance of considering autoimmunity and inflammatory features in addition to hypogammaglobulinemia and recurrent infections. Moreover, the different performance in terms of sensitivity of the “10 Warning Signs” by the Jeffrey Modell Foundation was highlighted, suggesting the diagnostic parameters used for CVID need to be different between pediatric and adult patients (i.e., phenotypes of inflammation, autoimmunity, malignancy, and atopy) [46]. Implementing ML tools could significantly reduce diagnostic delays, leading to earlier and more effective treatments. With more ML studies, such nuanced differences between diagnostics and treatment strategies may become more apparent as new patterns are recognized that have not been noted before. Advanced ML-based algorithms can identify patterns within large-scale EHR data, enabling them to predict patient diagnoses and outcomes. These algorithms can assist early detection by integrating information from multiple sources and support clinical decision-making for diagnosis and interventions [41]. SPIRIT analyzer, a rule-based AI tool (i.e., a computer system in which domain-specific knowledge is represented in the form of rules and general-purpose reasoning is used to solve problems in the domain), demonstrated that the presence of two or more warning signs was predictive of IEI in respect of healthy subjects [20,45,63] Notably, Rider et al. proved that bronchiectasis, splenomegaly, and particular infections related to pneumonia, such as *Streptococcus pneumoniae* and interstitial pneumonia, showed greater specificity for IEI [27]. Therefore, the study provides additional stratification for population care logistics, aiming to reduce the number of referrals. This could serve as a practical application of ML in managing patient workloads at the clinic. Taken together, these new efforts have moved beyond the 10 Warning Signs and can successfully identify patients with IEIs from their EHR [49].

The application of ML and AI has been mainly limited to analyzing structured EHR, laboratory, and genomic data. Current approaches typically begin by ‘training’ an AI algorithm to recognize IEI based on the clinical features of known cases, a method similar to our current approaches to training medical students, residents, and fellows about IEI, which involve repeatedly exposing them to actual cases [49]. Still, using unstructured data (i.e., free text) remains largely untapped as a resource [20,27,36,41]. NLP is a promising approach to address this limitation. Specifically, NLP refers to the branch of computer science and AI that represents free text in a manner that makes it interpretable by computers. Roberts et al. showed that sufficient information exists within clinical notes to make an early diagnosis. A prediction model can detect diseases before diagnosis using current clinical workflows [44]. Future research could leverage NLP to systematically extract and structure relevant diagnostic information from unstructured clinical notes. NLP pipelines can be designed to capture IEI-relevant phenotypic cues from these notes, considering: (i) domain-specific terminology, abbreviations, and negations; (ii) integration of extracted features with structured EHR and laboratory data; (iii) validation of algorithms against gold-standard, clinician-annotated datasets. Furthermore, addressing challenges such as bias, privacy, and generalizability across healthcare settings would provide valuable guidance for advancing this field.

Various computational techniques for analyzing flow cytometry data have been recently developed (e.g., flowAI and flowClean), automating the steps in the data analysis pipeline to make data analysis reproducible [64,65,66]. These techniques can automatically evaluate scatter and marker values over time and filter out regions that show abnormal behavior. AI-assisted tools, such as flowSOM and DeepFlow, were employed for flow cytometry in patients with CVID and other immune disorders [17,36].

BN is a probabilistic graphical model that embeds data, expert opinion, or both into an intuitive graph, enabling reasoning in the presence of inherent uncertainty [67,68]. BN-based tools have demonstrated their ability and accuracy in decision-making, improving diagnostic rates for IEI in patients with recurrent infections and predicting T-cell reconstitution post-transplant. This highlights how predictive analytics can personalize therapeutic strategies [23,38].

Gradient boosting was developed and optimized for classification and prediction, offering improved interpretability and performance, even in cases where a small training dataset is available and contains many features. Additionally, it proves effective in handling missing values, skewness, and outliers, and making automatic decisions [18]. XGBoost is a robust boosting-trees-based supervised ML algorithm that accurately predicts IEI [69]. Wang et al. use this algorithm to reliably predict early mortality after UCBT in patients with IEI, while Méndez Barrera et al. leverage it to identify clinical and laboratory features to predict several IEI [18,43].

The main benefit of Enet is the prevention of overfitting. Sobrino et al. identified IFN genes and transcription factors as predictive markers of HSC engraftment failure in patients with CGD [47].

LR is a widely used ML approach. It is simple and interpretable, assuming a linear dependence between the log odds of the outcome and predictors [41]. Starting from its wide use in radiomics, LR-based tools were used to predict immunodeficiency in children with pulmonary tuberculosis, demonstrating the potential of combining AI and radiological data even to detect underlying IEI [42].

Training AI still presents challenges due to the limited availability of data, particularly for rare diseases, overlapping phenotypes, and biases present in current datasets. Age and sex introduce biases into training data; therefore, comparing the effectiveness of different AI techniques is difficult. Moreover, many ethical considerations and challenges play a significant role in the deployment of AI (privacy and data security; biases in the design of an AI model that may lead to discrimination; ethical decision making in the uses of AI; concerns related to transparency, accountability, and data governance) [49]. AI could also generate misinterpretations that require a domain-specific error handling and iterative model refinement to ensure clinical reliability. For instance, using an NLP SVM-based algorithm that interprets text notes, there is a risk of false positives when medical abbreviations or acronyms overlap with common words (e.g., ‘was’ is misinterpreted as ‘WAS’ gene) [44]. Refining the training corpus, adding domain-specific term disambiguation rules, and integrating contextual cues from surrounding text can mitigate it. Similarly, initial versions of phenotyping SVM [34] and gene-specific predictors [14] showed misclassification when compared with overlapping phenotypes or rare benign variants. These misinterpretations can be mitigated through refined feature engineering, the addition of training data, and the integration of functional validation.

Recent advances in AI methodology have been significant. However, the limited availability of data and the rarity and heterogeneity of IEI represent relevant challenges in developing models that can generalize effectively. Thus, the development of AI models for IEI requires aggregating data from various sources and applying expert clinical knowledge to improve feature selection [70]. Therefore, further limitations persist. Although the methodological rigor of the included studies was generally high, some needed more adequate reporting on sample size calculations, which could introduce bias. Some issues with these methods are also related to their reliance on structured EHR data, which leaves a potentially valuable resource, such as unstructured data, underutilized. Additionally, there is a need to integrate data from diverse sources and disease backgrounds to enhance model robustness and minimize the risk of selection bias in future studies. Despite the rigorous methodology, which ensured the inclusion of high-quality studies that provide reliable evidence, the variability in the quality of the included studies and the potential for selection bias may affect the generalizability of the findings. Moreover, future primary studies should strengthen their methodological rigor by including thorough sample size planning. Sample size calculations must be clearly justified and aligned with the study’s objectives and outcomes to improve validity and reproducibility. Transparent reporting of these parameters will help minimize the risk of studies being underpowered or overpowered, enhance reproducibility, and enable meaningful comparisons across different studies.

## 5. Conclusions

This review summarizes and provides insights into the application of ML in diagnosing IEI, with significant implications for improving diagnostic accuracy and personalizing patient care. Various ML approaches demonstrated high sensitivity and specificity in different clinical settings, confirming the potential of big data integration techniques to accurately diagnose complex conditions, including IEI, with significant improvement over conventional diagnostic methods. Future research should focus on validating the models in larger, more diverse populations and exploring their integration into clinical practice. While these models show promise in specialized settings, further work is needed to adapt and validate them in different clinical settings to ensure their utility across diverse patient populations. Moreover, future research may help define treatment recommendations for specific patients and situations, thus implementing precision and personalized healthcare.

## Figures and Tables

**Table 1:** A priori inclusion and exclusion criteria according to population (P), intervention (I), and outcome (O) (PIO) framework, and study design.

Inclusion Criteria	Exclusion Criteria
- Population: children and adult patients with a diagnosis of IEI according to Human IEI IUIS classification [1]. - Intervention: application of AI tools to support the diagnosis, prediction, and management of IEI, including the use of EHR, genetic/molecular data, clinical features, and flow cytometry data. - Outcome: improved diagnostic accuracy, early identification of IEI, prediction of disease outcomes, personalized treatment strategies, reduction in diagnostic time and healthcare costs, and enhanced understanding of molecular mechanisms. - Study design: original data (all study design).	- Clinical guidelines, consensus documents, reviews, systematic reviews, meta-analyses, abstracts, and conference proceedings. - Studies that did not involve the application of AI or ML models.

AI, artificial intelligence. EHR, electronic health records. IEI, inborn errors of immunity. IUIS, international union of immunological societies. ML, machine learning.

## Data Availability

The contributions presented in the study are included in the article, and further inquiries can be directed to the corresponding author (riccardo.castagnoli@unipv.it).

## Supplementary Materials

The following supporting information can be downloaded at: https://www.mdpi.com/article/10.3390/jcm14175958/s1. Table S1: PRISMA Checklist; Table S2: Summary of the main features of AI tools reported in the systematic review [71,72,73,74,75,76,77,78,79,80,81].

## References

[1] Tangye S.G., Al-Herz W., Bousfiha A., Cunningham-Rundles C., Franco J.L., Holland S.M., Klein C., Morio T., Oksenhendler E., Picard C. (2022). Human Inborn Errors of Immunity: 2022 Update on the Classification from the International Union of Immunological Societies Expert Committee. J. Clin. Immunol..

[2] Casanova J.L., Abel L. (2013). The Genetic Theory of Infectious Diseases: A Brief History and Selected Illustrations. Annu. Rev. Genom. Hum. Genet..

[3] Casanova J.L., Abel L. (2020). The human genetic determinism of life-threatening infectious diseases: Genetic heterogeneity and physiological homogeneity?. Hum. Genet..

[4] Notarangelo L.D., Bacchetta R., Casanova J.L., Su H.C. (2020). Human inborn errors of immunity: An expanding universe. Sci. Immunol..

[5] Castagnoli R., Lougaris V., Giardino G., Volpi S., Leonardi L., La Torre F., Federici S., Corrente S., Cinicola B.L., Soresina A. (2021). Allergy ITF of the IS of P, Immunology (SIAIP). Inborn errors of immunity with atopic phenotypes: A practical guide for allergists. World Allergy Organ. J..

[6] Sogkas G., Witte T. (2023). The link between rheumatic disorders and inborn errors of immunity. eBioMedicine.

[7] Taietti I., Catamerò F., Lodi L., Giovannini M., Castagnoli R. (2025). Inborn errors of immunity with atopic phenotypes in the allergy and immunology clinic: A practical review. Curr. Opin. Allergy Clin. Immunol..

[8] Castagnoli R., Cenzato F., Taietti I., Licari A., Marseglia G.L. (2025). Studying inborn errors of immunity to understand the pathogenic mechanisms underlying highly prevalent immune-mediated diseases. Minerva Pediatr..

[9] Staels F., Collignon T., Betrains A., Gerbaux M., Willemsen M., Humblet-Baron S., Liston A., Vanderschueren S., Schrijvers R. (2021). Monogenic Adult-Onset Inborn Errors of Immunity. Front. Immunol..

[10] Fischer A. (2023). Gene therapy for inborn errors of immunity: Past, present and future. Nat. Rev. Immunol..

[11] Abolhassani H., Azizi G., Sharifi L., Yazdani R., Mohsenzadegan M., Delavari S., Sohani M., Shirmast P., Chavoshzadeh Z., Mahdaviani S.A. (2020). Global systematic review of primary immunodeficiency registries. Expert. Rev. Clin. Immunol..

[12] Rider N.L., Srinivasan R., Khoury P. (2020). Artificial intelligence and the hunt for immunological disorders. Curr. Opin. Allergy Clin. Immunol..

[13] Lee K., Abraham R.S. (2021). Next-generation sequencing for inborn errors of immunity. Human. Immunol..

[14] Fang M., Su Z., Abolhassani H., Itan Y., Jin X., Hammarström L. (2022). VIPPID: A gene-specific single nucleotide variant pathogenicity prediction tool for primary immunodeficiency diseases. Brief. Bioinform..

[15] Casanova J.L. (2015). Severe infectious diseases of childhood as monogenic inborn errors of immunity. Proc. Natl. Acad. Sci. USA.

[16] Modell V., Quinn J., Ginsberg G., Gladue R., Orange J., Modell F. (2017). Modeling strategy to identify patients with primary immunodeficiency utilizing risk management and outcome measurement. Immunol. Res..

[17] Lu Z., Morita M., Yeager T.S., Lyu Y., Wang S.Y., Wang Z., Fan G. (2024). Validation of Artificial Intelligence (AI)-Assisted Flow Cytometry Analysis for Immunological Disorders. Diagnostics.

[18] Barrera J.A.M., Guzmán S.R., Cascajares E.H., Garabedian E.K., Fuleihan R.L., Sullivan K.E., Reyes S.O.L. (2023). Who’s your data? Primary immune deficiency differential diagnosis prediction via machine learning and data mining of the USIDNET registry. Clin. Immunol..

[19] Modell V., Quinn J., Orange J., Notarangelo L.D., Modell F. (2016). Primary immunodeficiencies worldwide: An updated overview from the Jeffrey Modell Centers Global Network. Immunol. Res..

[20] Modell V., Gee B., Lewis D.B., Orange J.S., Roifman C.M., Routes J.M., Sorensen R.U., Notarangelo L.D., Modell F. (2011). Global study of primary immunodeficiency diseases (PI)—Diagnosis, treatment, and economic impact: An updated report from the Jeffrey Modell Foundation. Immunol. Res..

[21] Arai Y., Kondo T., Fuse K., Shibasaki Y., Masuko M., Sugita J., Teshima T., Uchida N., Fukuda T., Kakihana K. (2019). Using a machine learning algorithm to predict acute graft-versus-host disease following allogeneic transplantation. Blood Adv..

[22] Shouval R., Labopin M., Unger R., Giebel S., Ciceri F., Schmid C., Esteve J., Baron F., Gorin N.C., Savani B. (2016). Prediction of Hematopoietic Stem Cell Transplantation Related Mortality- Lessons Learned from the In-Silico Approach: A European Society for Blood and Marrow Transplantation Acute Leukemia Working Party Data Mining Study. PLoS ONE.

[23] Rider N.L., Cahill G., Motazedi T., Wei L., Kurian A., Noroski L.M., Seeborg F.O., Chinn I.K., Roberts K., Son L.H. (2021). PI Prob: A risk prediction and clinical guidance system for evaluating patients with recurrent infections. PLoS ONE.

[24] Knight V., Heimall J.R., Chong H., Nandiwada S.L., Chen K., Lawrence M.G., Akha A.A.S., Kumánovics A., Jyonouchi S., Ngo S.Y. (2021). A Toolkit and Framework for Optimal Laboratory Evaluation of Individuals with Suspected Primary Immunodeficiency. J. Allergy Clin. Immunol. Pract..

[25] Khoury P., Srinivasan R., Kakumanu S., Ochoa S., Keswani A., Sparks R., Rider N.L. (2022). A Framework for Augmented Intelligence in Allergy and Immunology Practice and Research—A Work Group Report of the AAAAI Health Informatics, Technology, and Education Committee. J. Allergy Clin. Immunol. Pract..

[26] Rider N.L. (2020). Digital systems for improving outcomes in patients with primary immune defects. Curr. Opin. Pediatr..

[27] Rider N.L., Coffey M., Kurian A., Quinn J., Orange J.S., Modell V., Modell F. (2023). A validated artificial intelligence-based pipeline for population-wide primary immunodeficiency screening. J. Allergy Clin. Immunol..

[28] Page M.J., McKenzie J.E., Bossuyt P.M., Boutron I., Hoffmann T.C., Mulrow C.D., Shamseer L., Tetzlaff J.M., Akl E.A., Brennan S.E. (2021). The PRISMA 2020 statement: An updated guideline for reporting systematic reviews. BMJ.

[29] Wolff R.F., Moons K.G., Riley R., Whiting P.F., Westwood M., Collins G.S., Reitsma J.B., Kleijnen J., Mallett S., The PROBAST Group (2019). PROBAST: A Tool to Assess the Risk of Bias and Applicability of Prediction Model Studies. Ann. Intern. Med..

[30] Collins G.S., Dhiman P., Navarro C.L.A., Ma J., Hooft L., Reitsma J.B., Logullo P., Beam A.L., Peng L., Van Calster B. (2021). Protocol for development of a reporting guideline (TRIPOD-AI) and risk of bias tool (PROBAST-AI) for diagnostic and prognostic prediction model studies based on artificial intelligence. BMJ Open.

[31] Keerthikumar S., Bhadra S., Kandasamy K., Raju R., Ramachandra Y., Bhattacharyya C., Imai K., Ohara O., Mohan S., Pandey A. (2009). Prediction of Candidate Primary Immunodeficiency Disease Genes Using a Support Vector Machine Learning Approach. DNA Res..

[32] Woellner C., Gertz E.M., Schäffer A.A., Lagos M., Perro M., Glocker E.-O., Pietrogrande M.C., Cossu F., Franco J.L., Matamoros N. (2010). Mutations in STAT3 and diagnostic guidelines for hyper-IgE syndrome. J. Allergy Clin. Immunol..

[33] Orange J.S., Glessner J.T., Resnick E., Sullivan K.E., Lucas M., Ferry B., Kim C.E., Hou C., Wang F., Chiavacci R. (2011). Genome-wide association identifies diverse causes of common variable immunodeficiency. J. Allergy Clin. Immunol..

[34] Engelhardt K.R., Gertz M.E., Keles S., Schäffer A.A., Sigmund E.C., Glocker C., Saghafi S., Pourpak Z., Ceja R., Sassi A. (2015). The extended clinical phenotype of 64 patients with dedicator of cytokinesis 8 deficiency. J. Allergy Clin. Immunol..

[35] Mücke U., Klemann C., Baumann U., Meyer-Bahlburg A., Kortum X., Klawonn F., Lechner W.M., Grigull L. (2017). Patient’s Experience in Pediatric Primary Immunodeficiency Disorders: Computerized Classification of Questionnaires. Front. Immunol..

[36] Emmaneel A., Bogaert D.J., Van Gassen S., Tavernier S.J., Dullaers M., Haerynck F., Saeys Y. (2019). A Computational Pipeline for the Diagnosis of CVID Patients. Front. Immunol..

[37] Berbers R.-M., Drylewicz J., Ellerbroek P.M., van Montfrans J.M., Dalm V.A.S.H., van Hagen P.M., Keller B., Warnatz K., van de Ven A., van Laar J.M. (2021). Targeted Proteomics Reveals Inflammatory Pathways that Classify Immune Dysregulation in Common Variable Immunodeficiency. J. Clin. Immunol..

[38] Diana J.-S., Bouazza N., Couzin C., Castelle M., Magnani A., Magrin E., Rosain J., Treluyer J.-M., Picard C., Moshous D. (2022). Bayesian Modeling Immune Reconstitution Apply to CD34+ Selected Stem Cell Transplantation for Severe Combined Immunodeficiency. Front. Pediatr..

[39] Abyazi M.L., Bell K.A., Gyimesi G., Baker T.S., Byun M., Ko H.M., Cunningham-Rundles C., Feng F., Maglione P.J. (2022). Convergence of cytokine dysregulation and antibody deficiency in common variable immunodeficiency with inflammatory complications. J. Allergy Clin. Immunol..

[40] Takao M.M.V., Carvalho L.S.F., Silva P.G.P., Pereira M.M., Viana A.C., da Silva M.T.N., Riccetto A.G.L. (2022). Artificial Intelligence in Allergy and Immunology: Comparing Risk Prediction Models to Help Screen Inborn Errors of Immunity. Int. Arch. Allergy Immunol..

[41] Mayampurath A., Ajith A., Anderson-Smits C., Chang S.-C., Brouwer E., Johnson J., Baltasi M., Volchenboum S., Devercelli G., Ciaccio C.E. (2022). Early Diagnosis of Primary Immunodeficiency Disease Using Clinical Data and Machine Learning. J. Allergy Clin. Immunol. Pract..

[42] Ding H., Chen X., Wang H., Zhang L., Wang F., He L. (2023). Identifying immunodeficiency status in children with pulmonary tuberculosis: Using radiomics approach based on un-enhanced chest computed tomography. Transl. Pediatr..

[43] Wang P., Liu C., Wei Z., Jiang W., Sun H., Wang Y., Hou J., Sun J., Huang Y., Wang H. (2023). Nomogram for Predicting Early Mortality after Umbilical Cord Blood Transplantation in Children with Inborn Errors of Immunity. J. Clin. Immunol..

[44] Roberts K., Chin A.T., Loewy K., Pompeii L., Shin H., Rider N.L. (2024). Natural language processing of clinical notes enables early inborn error of immunity risk ascertainment. J. Allergy Clin. Immunol. Glob..

[45] Rider N.L., Truxton A., Ohrt T., Margolin-Katz I., Horan M., Shin H., Davila R., Tenembaum V., Quinn J., Modell V. (2024). Validating inborn error of immunity prevalence and risk with nationally representative electronic health record data. J. Allergy Clin. Immunol..

[46] Johnson R., Stephens A.V., Mester R., Knyazev S., Kohn L.A., Freund M.K., Bondhus L., Hill B.L., Schwarz T., Zaitlen N. (2024). Electronic health record signatures identify undiagnosed patients with common variable immunodeficiency disease. Sci. Transl. Med..

[47] Sobrino S., Magnani A., Semeraro M., Martignetti L., Cortal A., Denis A., Couzin C., Picard C., Bustamante J., Magrin E. (2023). Severe hematopoietic stem cell inflammation compromises chronic granulomatous disease gene therapy. Cell Rep. Med..

[48] Pello E., Kainulainen L., Vakkilainen M., Klemetti P., Taskinen M., Mäkitie O., Vakkilainen S. (2024). Shorter birth length and decreased T-cell production and function predict severe infections in children with non–severe combined immunodeficiency cartilage–hair hypoplasia. J. Allergy Clin. Immunol. Glob..

[49] Rivière J.G., Palacín P.S., Butte M.J. (2024). Proceedings from the inaugural Artificial Intelligence in Primary Immune Deficiencies (AIPID) conference. J. Allergy Clin. Immunol..

[50] Akalu Y.T., Bogunovic D. (2024). Inborn errors of immunity: An expanding universe of disease and genetic architecture. Nat. Rev. Genet..

[51] Odnoletkova I., Kindle G., Quinti I., Grimbacher B., Knerr V., Gathmann B., Ehl S., Mahlaoui N., Van Wilder P., Bogaerts K. (2018). The burden of common variable immunodeficiency disorders: A retrospective analysis of the European Society for Immunodeficiency (ESID) registry data. Orphanet J. Rare Dis..

[52] Khanna N.N., Maindarkar M.A., Viswanathan V., E Fernandes J.F., Paul S., Bhagawati M., Ahluwalia P., Ruzsa Z., Sharma A., Kolluri R. (2022). Economics of Artificial Intelligence in Healthcare: Diagnosis vs. Treatment. Healthcare.

[53] Park K.J., Gromiha M.M., Horton P., Suwa M. (2005). Discrimination of outer membrane proteins using support vector machines. Bioinformatics.

[54] Prashanth R., Dutta Roy S., Mandal P.K., Ghosh S. (2016). High-Accuracy Detection of Early Parkinson’s Disease through Multimodal Features and Machine Learning. Int. J. Med. Inform..

[55] Choi E., Schuetz A., Stewart W.F., Sun J. (2017). Using recurrent neural network models for early detection of heart failure onset. J. Am. Med. Inform. Assoc..

[56] Costa L., Gago M.F., Yelshyna D., Ferreira J., Silva H.D., Rocha L., Sousa N., Bicho E. (2016). Application of Machine Learning in Postural Control Kinematics for the Diagnosis of Alzheimer’s Disease. Comput. Intell. Neurosci..

[57] Taslimitehrani V., Dong G., Pereira N.L., Panahiazar M., Pathak J. (2016). Developing EHR-driven heart failure risk prediction models using CPXR(Log) with the probabilistic loss function. J. Biomed. Inform..

[58] Lingren T., Chen P., Bochenek J., Doshi-Velez F., Manning-Courtney P., Bickel J., Welchons L.W., Reinhold J., Bing N., Ni Y. (2016). Electronic Health Record Based Algorithm to Identify Patients with Autism Spectrum Disorder. PLoS ONE.

[59] Lyman J.A., Cohn W.F., Bloomrosen M., Detmer D.E. (2010). Clinical decision support: Progress and opportunities. J. Am. Med. Inform. Assoc..

[60] Bates D.W., Kuperman G.J., Wang S., Gandhi T., Kittler A., Volk L., Spurr C., Khorasani R., Tanasijevic M., Middleton B. (2003). Ten Commandments for Effective Clinical Decision Support: Making the Practice of Evidence-based Medicine a Reality. J. Am. Med. Inform. Assoc. JAMIA.

[61] Vandewiele G., De Backere F., Lannoye K., Berghe M.V., Janssens O., Van Hoecke S., Keereman V., Paemeleire K., Ongenae F., De Turck F. (2018). A decision support system to follow up and diagnose primary headache patients using semantically enriched data. BMC Med. Inform. Decis. Mak..

[62] Cahan A., Cimino J.J. (2017). A Learning Health Care System Using Computer-Aided Diagnosis. J. Med. Internet Res..

[63] Rider N.L., Miao D., Dodds M., Modell V., Modell F., Quinn J., Schwarzwald H., Orange J.S. (2019). Calculation of a Primary Immunodeficiency “Risk Vital Sign” via Population-Wide Analysis of Claims Data to Aid in Clinical Decision Support. Front. Pediatr..

[64] Saeys Y., Van Gassen S., Lambrecht B.N. (2016). Computational flow cytometry: Helping to make sense of high-dimensional immunology data. Nat. Rev. Immunol..

[65] Monaco G., Chen H., Poidinger M., Chen J., de Magalhães J.P., Larbi A. (2016). flowAI: Automatic and interactive anomaly discerning tools for flow cytometry data. Bioinformatics.

[66] Fletez-Brant K., Špidlen J., Brinkman R.R., Roederer M., Chattopadhyay P.K. (2016). flowClean: Automated identification and removal of fluorescence anomalies in flow cytometry data. Cytom. Part A.

[67] Arora P., Boyne D., Slater J.J., Gupta A., Brenner D.R., Druzdzel M.J. (2019). Bayesian Networks for Risk Prediction Using Real-World Data: A Tool for Precision Medicine. Value Health.

[68] Korb K.B., Nicholson A.E. (2010). Bayesian Artificial Intelligence.

[69] Vega J.B.M. Tutorial: XGBoost en Python. Medium. 24 January 2022. https://medium.com/@jboscomendoza/tutorial-xgboost-en-python-53e48fc58f73.

[70] Martinson A.K., Chin A.T., Butte M.J., Rider N.L. (2024). Artificial Intelligence and Machine Learning for Inborn Errors of Immunity: Current State and Future Promise. J. Allergy Clin. Immunol. Pract..

[71] Noble W. (2006). What is a support vector machine?. Nat. Biotechnol..

[72] Breiman L. (2001). Random Forests. Mach. Learn..

[73] Marimuthu R., Shivappriya S.N., Saroja M.N., Jude H.D. (2021). Chapter 14—A study of machine learning algorithms used for detecting cognitive disorders associated with dyslexia. Handbook of Decision Support Systems for Neurological Disorders.

[74] Jurafsky D., Martin J.H. Speech and Language Processing—Chapter 5. https://pages.ucsd.edu/~bakovic/compphon/Jurafsky,%20Martin.-Speech%20and%20Language%20Processing_%20An%20Introduction%20to%20Natural%20Language%20Processing%20(2007).pdf.

[75] ElKalaawy N., Wassal A. (2015). Methodologies for the modeling and simulation of biochemical networks, illustrated for signal transduction pathways: A primer. Biosystems.

[76] Sara van Erp Daniel L. (2019). Oberski, Joris Mulder, Shrinkage priors for Bayesian penalized regression. J. Math. Psychol..

[77] Liu J., Ma Y., Xie W., Li X., Wang Y., Xu Z., Bai Y., Yin P., Wu Q. (2023). Lasso-Based Machine Learning Algorithm for Predicting Postoperative Lung Complications in Elderly: A Single-Center Retrospective Study from China. Clin. Interv. Aging.

[78] Zhang W., Gu X., Hong L., Han L., Wang L. (2023). Comprehensive review of machine learning in geotechnical reliability analysis: Algorithms, applications and further challenges. Appl. Soft. Comput..

[79] Díez-Sanmartín C., Sarasa-Cabezuelo A., Belmonte A.A. (2021). The impact of artificial intelligence and big data on end-stage kidney disease treatments. Expert Syst. Appl..

[80] Krzywinski M., Altman N. (2017). Classification and regression trees. Nat. Methods.

[81] Pettit R.W., Fullem R., Cheng C., Amos C.I. (2021). Artificial intelligence, machine learning, and deep learning for clinical outcome prediction. Emerg. Top. Life Sci..

