# Supplementary material for: Application of Artificial Intelligence in Inborn Errors of Immunity Identification and Management: Past, Present, and Future—A Systematic Review
Source: J Clin Med. 2025 Aug 23;14(17):5958. doi: 10.3390/jcm14175958 (PMC12429086; doi:10.3390/jcm14175958)
Supplement: Supplementary file 1 [file jcm-14-05958-s001.zip › jcm-3769560-supplementary.pdf]

Journal of Clinical Medicine

MDPI

Table S1. PRISMA Checklist.

Section and Topic	Item #	Checklist item	Location where item is reported
**TITLE**			
Title	1	Identify the report as a systematic review.	1
**ABSTRACT**			
Abstract	2	See the PRISMA 2020 for Abstracts checklist.	1
**INTRODUCTION**			
Rationale	3	Describe the rationale for the review in the context of existing knowledge.	2,3
Objectives	4	Provide an explicit statement of the objective(s) or question(s) the review addresses.	3
**METHODS**			
Eligibility criteria	5	Specify the inclusion and exclusion criteria for the review and how studies were grouped for the syntheses.	3
Information sources	6	Specify all databases, registers, websites, organisations, reference lists and other sources searched or consulted to identify studies. Specify the date when each source was last searched or consulted.	3
Search strategy	7	Present the full search strategies for all databases, registers and websites, including any filters and limits used.	3
Selection process	8	Specify the methods used to decide whether a study met the inclusion criteria of the review, including how many reviewers screened each record and each report retrieved, whether they worked independently, and if applicable, details of automation tools used in the process.	3
Data collection process	9	Specify the methods used to collect data from reports, including how many reviewers collected data from each report, whether they worked independently, any processes for obtaining or confirming data from study investigators, and if applicable, details of automation tools used in the process.	3

Section and Topic	Item #	Checklist item	Location where item is reported
Data items	10a	List and define all outcomes for which data were sought. Specify whether all results that were compatible with each outcome domain in each study were sought (e.g. for all measures, time points, analyses), and if not, the methods used to decide which results to collect.	3
	10b	List and define all other variables for which data were sought (e.g. participant and intervention characteristics, funding sources). Describe any assumptions made about any missing or unclear information.	3
Study risk of bias assessment	11	Specify the methods used to assess risk of bias in the included studies, including details of the tool(s) used, how many reviewers assessed each study and whether they worked independently, and if applicable, details of automation tools used in the process.	4
Effect measures	12	Specify for each outcome the effect measure(s) (e.g. risk ratio, mean difference) used in the synthesis or presentation of results.	3,4
Synthesis methods	13a	Describe the processes used to decide which studies were eligible for each synthesis (e.g. tabulating the study intervention characteristics and comparing against the planned groups for each synthesis (item #5)).	3,4
	13b	Describe any methods required to prepare the data for presentation or synthesis, such as handling of missing summary statistics, or data conversions.	3,4
	13c	Describe any methods used to tabulate or visually display results of individual studies and syntheses.	3,4
	13d	Describe any methods used to synthesize results and provide a rationale for the choice(s). If meta-analysis was performed, describe the model(s), method(s) to identify the presence and extent of statistical heterogeneity, and software package(s) used.	3,4
	13e	Describe any methods used to explore possible causes of heterogeneity among study results (e.g. subgroup analysis, meta-regression).	3,4
	13f	Describe any sensitivity analyses conducted to assess robustness of the synthesized results.	3,4
Reporting bias assessment	14	Describe any methods used to assess risk of bias due to missing results in a synthesis (arising from reporting biases).	4
Certainty assessment	15	Describe any methods used to assess certainty (or confidence) in the body of evidence for an outcome.	Not applicable
**RESULTS**			
Study selection	16a	Describe the results of the search and selection process, from the number of records identified in the search to the number of studies	4-5

Section and Topic	Item #	Checklist item	Location where item is reported
		included in the review, ideally using a flow diagram.	
	16b	Cite studies that might appear to meet the inclusion criteria, but which were excluded, and explain why they were excluded.	5-14
Study characteristics	17	Cite each included study and present its characteristics.	4-18
Risk of bias in studies	18	Present assessments of risk of bias for each included study.	4-18
Results of individual studies	19	For all outcomes, present, for each study: (a) summary statistics for each group (where appropriate) and (b) an effect estimate and its precision (e.g. confidence/credible interval), ideally using structured tables or plots.	4-18
Results of syntheses	20a	For each synthesis, briefly summarise the characteristics and risk of bias among contributing studies.	4-18
	20b	Present results of all statistical syntheses conducted. If meta-analysis was done, present for each the summary estimate and its precision (e.g. confidence/credible interval) and measures of statistical heterogeneity. If comparing groups, describe the direction of the effect.	4-18
	20c	Present results of all investigations of possible causes of heterogeneity among study results.	4-18
	20d	Present results of all sensitivity analyses conducted to assess the robustness of the synthesized results.	4-18
Reporting biases	21	Present assessments of risk of bias due to missing results (arising from reporting biases) for each synthesis assessed.	18
Certainty of evidence	22	Present assessments of certainty (or confidence) in the body of evidence for each outcome assessed.	Not applicable
**DISCUSSION**			
Discussion	23a	Provide a general interpretation of the results in the context of other evidence.	18-21
	23b	Discuss any limitations of the evidence included in the review.	18-21
	23c	Discuss any limitations of the review processes used.	21
	23d	Discuss implications of the results for practice, policy, and future research.	18-22

Section and Topic	Item #	Checklist item	Location where item is reported
**OTHER INFORMATION**			
Registration and protocol	24a	Provide registration information for the review, including register name and registration number, or state that the review was not registered.	3
	24b	Indicate where the review protocol can be accessed, or state that a protocol was not prepared.	3
	24c	Describe and explain any amendments to information provided at registration or in the protocol.	Not applicable
Support	25	Describe sources of financial or non-financial support for the review, and the role of the funders or sponsors in the review.	Not applicable
Competing interests	26	Declare any competing interests of review authors.	22
Availability of data, code and other materials	27	Report which of the following are publicly available and where they can be found: template data collection forms; data extracted from included studies; data used for all analyses; analytic code; any other materials used in the review.	22

From: Page MJ, McKenzie JE, Bossuyt PM, Boutron I, Hoffmann TC, Mulrow CD, et al. The PRISMA 2020 statement: an updated guideline for reporting systematic reviews. BMJ 2021;372:n71. doi: 10.1136/bmj.n71. This work is licensed under CC BY 4.0. To view a copy of this license, visit https://creativecommons.org/licenses/by/4.0/

Table S2. Summary of the main features of AI tools reported in the systematic review.

**AI tool**	
SVM (Supervised ML)	A computer algorithm that learns from examples to assign labels to objects by generating a classifier from positively and negatively labeled training datasets. It builds this classifier by mapping input samples into a potentially high-dimensional feature space [1].
RF (Supervised ML)	A classifier that uses a tree-like structure, with each tree built using independent feature vectors from random subsets of the input data. It combines the outputs of multiple decision trees—using majority voting for classification tasks or averaging for regression—to improve prediction accuracy and reduce overfitting[2,3].
LR (Supervised ML)	An algorithm used for classification tasks, where the goal is to predict the probability that an instance belongs to a given class. It is based a statistical method that models the relationship between input features and a binary outcome using a logistic function[4].
BN (Supervised/Unsupervised ML)	Probabilistic directed acyclic graph, where nodes represent variables and edges denote causal dependencies among them. These probabilistic graphical models incorporate data, expert knowledge, or both into an intuitive structure, allowing reasoning and inference under uncertainty[5].
Enet (ML)	A modified form of linear regression that uses the same hypothesis function for prediction. This regularized regression technique addresses issues such as multicollinearity among predictors and overfitting, which are common in high-dimensional datasets[6].
Lasso regression (ML)	Regularization technique that minimizes the potential collinearity of predictive variables and filters out the most influential ones applying a penalty to prevent overfitting and enhance the accuracy of statistical models[7].
XGboost (supervised ML)	A decision-tree-based ensemble method that employs regularization to control overfitting while predicting a response variable based on given covariates[8,9].
CART (supervised ML)	A decision tree algorithm that is used for both classification and regression tasks. It learns from labelled data to predict unseen data[10].
DNN (DL)	An artificial neural network in which each neuron in one layer is fully connected to every neuron in the next layer. This full connectivity forms enables learning complex relationships in the data[11].

AI: artificial intelligence; BN: Bayesian network; CART: classification and regression tree; DNN: dense neural network; DL: deep learning; Enet: elastic network regression; LR: logistic regression; L: machine learning; RF: random forest; SVM: support vector machine; XGboost: extreme gradient boosting.

References

1. Noble, W. What is a support vector machine? Nat. Biotechnol. 2006, 24, 1565–1567. https://doi.org/10.1038/nbt1206-1565.
2. Breiman, L. Random Forests. Mach. Learn. 2001, 45, 5–32. https://doi.org/10.1023/A:1010933404324.
3. Marimuthu, R.; Shivappriya, S.N.; Saroja, M.N. Chapter 14—A study of machine learning algorithms used for detecting cognitive disorders associated with dyslexia. In Jude, Handbook of Decision Support Systems for Neurological Disorders; Hemanth, D. Eds.; Academic Press: Cambridge, MA, USA, 2021; pp. 245–262; ISBN 9780128222713.
4. Jurafsky, D.; Martin, J.H. Speech and Language Processing—Chapter 5. Available online: https://pages.ucsd.edu/~bakovic/compphon/Jurafsky,%20Martin.-Speech%20and%20Language%20Processing_%20An%20Introduction%20to%20Natural%20Language%20Processing%20(2007).pdf (accessed on 15 February 2025).
5. ElKalaawy, N.; Wassal, A. Methodologies for the modeling and simulation of biochemical networks, illustrated for signal transduction pathways: a primer. Biosystems 2015, 129, 1–18. https://doi:10.1016/j.biosystems.2015.01.008.
6. Sara van Erp Daniel, L. Oberski, Joris Mulder, Shrinkage priors for Bayesian penalized regression. J. Math. Psychol. 2019, 89, 31–50; ISSN 0022-2496. https://doi.org/10.1016/j.jmp.2018.12.004.
7. Liu, J.; Ma, Y.; Xie, W.; Li, X.; Wang, Y.; Xu, Z.; Bai, Y.; Yin, P.; Wu, Q. Lasso-Based Machine Learning Algorithm for Predicting Postoperative Lung Complications in Elderly: A Single-Center Retrospective Study from China. Clin Interv Aging. 2023, 18, 597–606. https://doi.org/10.2147/CIA.S406735
8. Zhang, W.; Gu, X.; Hong, L.; Han, L.; Wang, L. Comprehensive review of machine learning in geotechnical reliability analysis: Algorithms, applications and further challenges. Applied. Soft. Computing 2023, 136, 110066. ISSN 1568-4946, https://doi.org/10.1016/j.asoc.2023.110066.
9. Díez-Sanmartín, C.; Sarasa-Cabezuelo, A.; Belmonte, A.A. The impact of artificial intelligence and big data on end-stage kidney disease treatments. Expert Syst. Appl. 2021, 180, 115076. ISSN 0957-4174. https://doi.org/10.1016/j.eswa.2021.115076.
10. Krzywinski, M., Altman, N. Classification and regression trees. Nat Methods 2017, 14, 757–758. https://doi.org/10.1038/nmeth.4370.
11. Pettit, R.W.; Fullem, R.; Cheng, C.; Amos, C.I. Artificial intelligence, machine learning, and deep learning for clinical outcome prediction. Emerg Top Life Sci. 2021, 5, 729–45. https://doi: 10.1042/ETLS20210246.

Disclaimer/Publisher's Note: The statements, opinions and data contained in all publications are solely those of the individual author(s) and contributor(s) and not of MDPI and/or the editor(s). MDPI and/or the editor(s) disclaim responsibility for any injury to people or property resulting from any ideas, methods, instructions or products referred to in the content.
